# Eliminating the interference of water for direct sensing of submerged plastics using hyperspectral near-infrared imager

**DOI:** 10.1038/s41598-023-39754-7

**Published:** 2023-10-06

**Authors:** Chunmao Zhu, Yugo Kanaya

**Affiliations:** Research Institute for Global Change, Japan Agency for Marine-Earth Science and Technology (JAMSTEC), Yokohama, Kanagawa 2360001 Japan

**Keywords:** Environmental sciences, Near-infrared spectroscopy

## Abstract

Interference from water in the reflectance spectra of plastics is a major obstacle to optical sensing of plastics in aquatic environments. Here we present evidence of the feasibility of sensing plastics in water using hyperspectral near-infrared to shortwave-infrared imaging techniques. We captured hyperspectral images of nine polymers submerged to four depths (2.5–15 mm) in water using a hyperspectral imaging system that utilizes near-infrared to shortwave-infrared light sources. We also developed algorithms to predict the reflectance spectra of each polymer in water using the spectra of the dry plastics and water as independent variables in a multiple linear regression model after a logarithmic transformation. A narrow 1100–1300 nm wavelength range was advantageous for detection of polyethylene, polystyrene, and polyvinyl chloride in water down to the 160–320 µm size range, while a wider 970–1670 nm wavelength range was beneficial for polypropylene reflectance spectrum prediction in water. Furthermore, we found that the spectra of the other five polymers, comprising polycarbonate, acrylonitrile butadiene styrene, phenol formaldehyde, polyacetal, and polymethyl methacrylate, could also be predicted within their respective optimized wavelength ranges. Our findings provide fundamental information for direct sensing of plastics in water on both benchtop and airborne platforms.

## Introduction

Along with the increasing consumption of plastics in the Anthropocene, mismanaged waste plastics have been entering both the freshwater and marine ecosystems in remarkable quantities. The amounts of macroplastics (≥ 5 mm) have been observed to increase significantly over time from 1957 to 2016, based on the recorded quantities that have been entangled with towed marine samplers^1^. In the future, the annual rates at which macro- and microplastics (< 5 mm) enter the ocean will increase 2.6-fold over the period from 2016 to 2040 if no measures are taken^2^, while the corresponding rate for microplastics would increase two-fold from 2016 to 2030 in the subtropical convergence zone^3^. Microplastics can be ingested by aquatic animals and retained in their vascular systems^4–7^, thus subsequently having an adverse effect on the food chain^8,9^. Macroplastic debris is harmful to marine mammals because it causes physical tangling and choking^10–12^. To provide a better understanding of the temporal and spatial distributions of plastics in both fresh water and the oceans, there is an urgent need to develop rapid detection techniques.

Hyperspectral imaging is a promising technique for detection of plastics based on their optical features^13–15^. Individual polymers have shown unique reflectance features in the near-infrared to shortwave-infrared (NIR-SWIR) wavelength range that are dependent on their C–H stretching overtones. When compared with current commonly-used methods for plastics detection, e.g., the Fourier transform infrared technique, the Raman spectral technique, and the pyrolysis gas-chromatography technique, hyperspectral imaging techniques are advantageous, with the lowest requirements for sample preprocessing and rapid detection speeds^16–20^. Moreover, encouraging results have been reported for detection of plastics with size ranges spanning from the micrometer to meter scales using hyperspectral imagers when applied over the range from benchtop to airborne and satellite platforms^21–24^. However, even such conceptual potential was proposed, technically it is still challenging to directly detect plastics submerged in water.

Hyperspectral imagers operating in the 900–2500 nm wavelength range have been basically applied to detection of plastics in the dry state using benchtop systems^18,19,25^. To provide a better cost performance, imager operation in the 900–1700 nm range is sufficient^24^. Important technical requirements that affect the detection capabilities of benchtop hyperspectral imaging systems on plastics in the dry state include the wavelength coverage of the illumination source, the photographic depth of field and exposure time, and the identification algorithms used^13,26^. In comparison, detection of plastics in the wet state is more demanding because of the interference from water in the spectra of the plastics, which are overlapped within the NIR-SWIR wavelength range. Over an extended (1000–2500 nm) wavelength range with use of well-conceived identification algorithms, plastics on wet filters were detected successfully^27^, although the detection capability was limited by the algorithms failing to separate the co-existing absorptive species in water.

Recent studies have attempted to establish hyperspectral reflectance datasets for a few polymers in the dry, wet, and submerged states^28–32^. Efforts have also been made to develop algorithms to eliminate interference from water when the plastics are submerged^30,33,34^. It was reported that a combination of the normalized vegetation difference index and the floating debris index has the potential to detect plastics in water using Sentinel-2 satellite observations^34,35^, although the plastic being even slightly submerged in the water would reduce its floating debris index toward zero^32^. There is thus still an urgent need to develop effective algorithms that can separate plastics from water^21,22,34^.

Differential optical absorption spectroscopy provides a powerful method to identify the specific absorption signatures of different materials^36^. The rationale behind this method is that scattered or direct incoming light spectra can be decomposed into the absorption contributions from multiple molecules that show overlapping absorption features using the Lambert–Beer law. In the atmospheric chemistry research field, the method has been applied successfully to measure the concentrations of different trace gases in the ambient air^37–40^. Using this method, the spectral features of soil and sand could be separated from atmospheric nitrogen dioxide based on measurements from the GOME-2 satellite^41^. Equivalent water thickness of land vegetation was derived from AVIRIS data applying the method along with spectrum-matching techniques^42^. Furthermore, in the aquatic environment, two phytoplankton groups, cyanobacteria, and diatoms showing distinct absorption features were also quantified successfully^43^. This principle could thus be applicable to separation of water and plastic absorptions within the same wavelength band.

**Figure 1:**
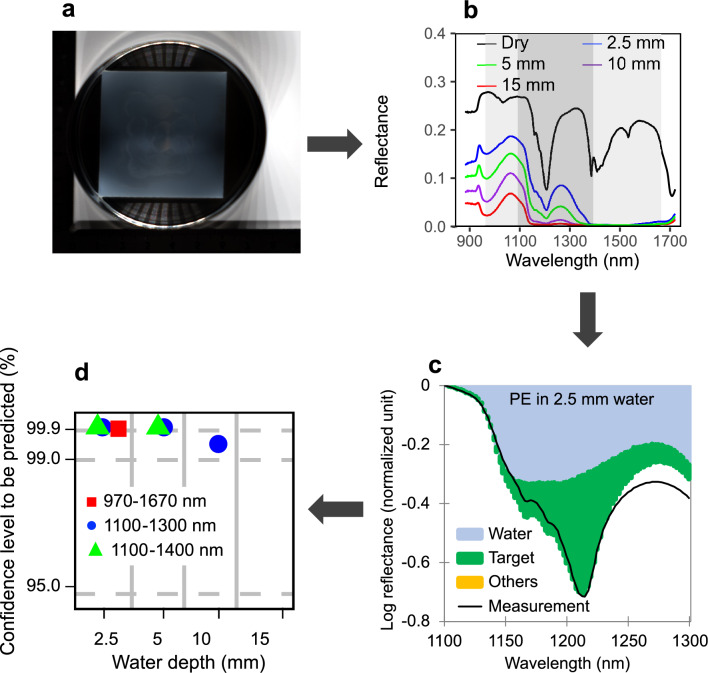
Schematic for eliminating the interference of water on the hyperspectral detection of submerged plastics. (**a**) Hyperspectral imageries of 9 polymers submerging in 4 water depths (2.5–15 mm) in the wavelength ranges of 900–1700 nm were acquired. Example of polyethylene is shown. (**b**) The reflectance spectra of a typical region of interests (an area of 16.0 × 16.0 mm^2^) covering submerged plastics were calculated. (**c**) The contributions from the target polymer, water and other polymers were separated using a multi-linear regression model after logarithmic transformation for polyethylene submerged in 2.5 mm depth water. (**d**) The capability to predict each of the submerged polymers was evaluated based on the confidence level of prediction.

In this work, we attempt to eliminate the interference caused by water from the detection of submerged plastics based on the principle of differential optical absorption spectroscopy using the NIR-SWIR hyperspectral imaging technique. The schematic of the procedures is shown in Fig. 1 and the system is shown in Fig. S1. We first obtained the reflectance spectra for plastics submerged in water (2.5 mm, 5 mm, 10 mm and 15 mm in depth) using a benchtop hyperspectral imaging system within the wavelength range from 900 to 1700 nm. Note that the measured signals fall in line with the terminology “transflectance”, which encompasses the reflection and absorption by the target (Fig. S1b). However, to keep with the conventional usage in the earth and environment field^31,32^, we are using “reflectance” in this study. Nine polymer types were investigated, including polyethylene (PE), polypropylene (PP), polystyrene (PS), polyvinyl chloride (PVC), polycarbonate (PC), acrylonitrile butadiene styrene (ABS), phenol formaldehyde (PF), polyacetal (POM) and polymethyl methacrylate (PMMA). Previous works suggested that completely dry conditions were favored for detection of plastics when using the hyperspectral imaging technique^24^. In this work, however, we aim to apply the method to detection of plastics in the wet state and under floating conditions on the sea surface when covered by thin water layers. We then developed algorithms to separate the contribution of water to the reflectance spectra of the submerged plastics by applying a multiple linear regression model after a logarithmic transformation. Subsequently, we evaluated the predictability of the submerged plastics regarding the polymer types, their size ranges, and the water depths, and considered wavelength range optimization. Our findings demonstrated the feasibility of applying hyperspectral imaging techniques directly to detection of plastics in surface water.

## Results

### Reflectance spectra of nine polymers in water

**Figure 2:**
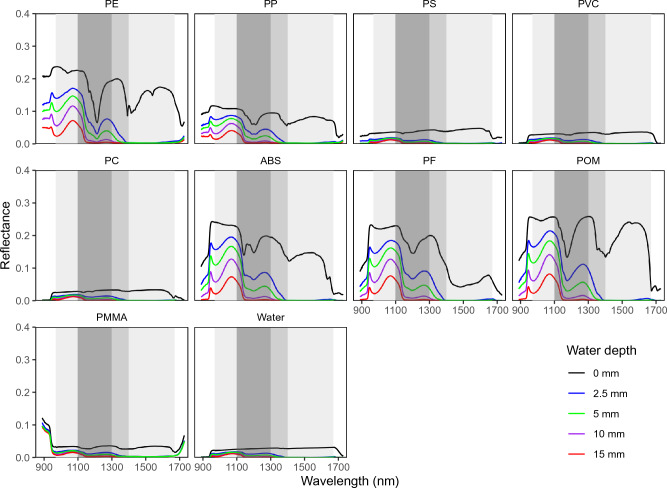
Spectra of plastics when submerged in water. Mean spectra on an area of 100 × 100 pixels of nine polymers submerged at four water depths of 2.5 mm, 5 mm, 10 mm, and 15 mm are shown. The shaded wavelength ranges of 970–1670 nm, 1100–1300 nm, and 1100–1400 nm were screened out for further prediction of the spectra of plastics in water based on the spectra of these plastics in the dry state (0 mm) and that of water.

Although plastics showed featured reflectance spectra in the dry state, they were notably affected by the absorbance of water when submerged (Fig. 2). In the dry state, the plastics absorbed photons at wavelengths centered on the 1100–1400 nm and 1600–1670 nm ranges. In comparison, as the wavelength increased, the reflectance spectrum of water showed notable dragging downward in both the 1130–1150 nm range and the 1300–1400 nm range. In the 1400–1700 nm range, the reflectance of water is close to zero. This high light absorption by water within the NIR-SWIR range interfered substantially with the reflectance signals of the plastics when they were submerged. Specifically, the featured reflectance spectra of PP and ABS in the dry state in the 1390–1410 nm range were hardly noticeable when these polymers were submerged in water.

As the water depth increased, the spectral features of the plastics were weakened, in agreement with previous studies^29,32^. At water depths of 10 mm and 15 mm, the reflectance spectra of most of these polymers were largely flattened. Garaba and Harmel reported that the reflectance spectra of PP at 490 nm and 860 nm were smoothed away at water depths greater than 0.32 m^30^. In comparison, our results indicate that this phenomenon occurred at the relatively longer wavelengths (NIR and SWIR) at shallower depths, i.e., on the millimeter scale, for pure water. Our results agreed with Moshtaghi et al., who found that the featured absorption of PP at 1070 nm could not be caught when the plastics were submerged by more than 50 mm^32^. Nevertheless, depending on the individual polymers, some featured spectra remained within specific wavelength ranges when the plastics were submerged in water. Therefore, we examined the possibility of predicting the spectra of plastics when submerged in water further.

### Predictability of four common polymers in water

**Figure 3:**
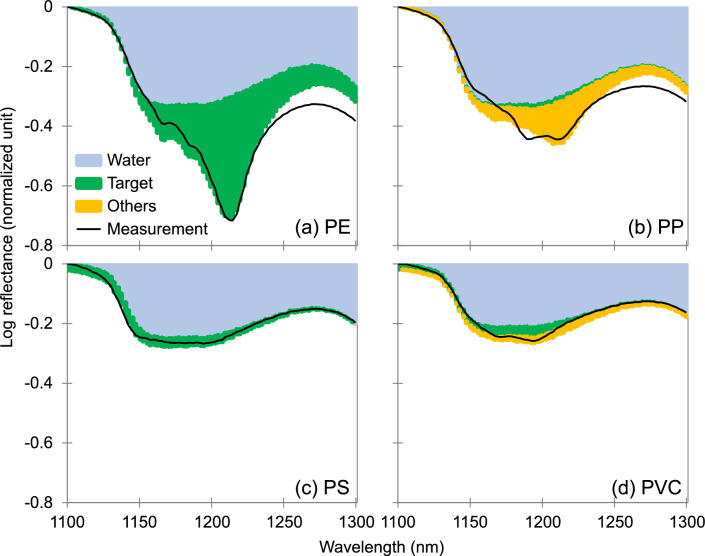
Separated contributions to the spectra of four common submerged polymers. Reflectance spectra of (**a**) PE, (**b**) PP, (**c**) PS, and (**d**) PVC in 2.5 mm of water were composed of contributions from the target polymer, the other polymers, and water, and were compared with the measurements. The logarithmic reflectance obtained after normalization was performed by subtracting the maximum value for each polymer in the 1100–1300 nm wavelength range on an area of 100 × 100 pixels are shown.

Of different polymers, polyethylene (PE), polypropylene (PP), polystyrene (PS) and polyvinyl chloride (PVC) contributed to 61% of global primary plastic wastes in 2015^44^. Therefore, we first investigated these 4 most common polymers, on the predictability of reflectance spectra of plastics in water by linear combination of plastics in the dry state and water, respectively. To do that, the observed reflectance spectra was first simulated using a linear regression model, where the contributions from each variable (water and polymers) were calculated. Parameters relating to these contributions were used to evaluate the predictability. In the 1100–1300 nm wavelength range on an area of 100 × 100 pixels (16.0 × 16.0 mm^2^), the prediction parameters for the spectra of PE, PP, PS, and PVC when submerged to a depth of 2.5 mm in water were summarized in Table 1. Meanwhile, their spectra were predicted as the sum of the contributions from three components: the target polymer, the other polymers, and water (Fig. 3). The spectra of PE, PS and PVC when submerged to a depth of 2.5 mm in water were predominantly driven by the spectrum of the target polymer in the dry state from among the other polymer spectra (*p* < 0.001) (i.e., PE in the dry state for the prediction of PE in water, see Table 1). Although the inevitable interference from water was present (light blue shades in Fig. 3), the featured spectra of the polymers in water for PE, PS, and PVC were reproduced well. This was attributed to the featured spectra of each target polymer (green shades in Fig. 3).

**Table 1:** Prediction parameters for four common polymers in 2.5-mm-deep water in the 1100–1300 nm wavelength range on an area of 100 × 100 pixels.

Target polymer	Driving variable	Coefficient	*p* value	Confidence level (*c*, %)
PE	Intercept	0 ± 0.02*	1	0
	PE	0.75 ± 0.12	< 0.001	> 99.9
	PP	0 ± 0.33	1	0
	PS	0 ± 0.32	1	0
	PVC	0 ± 0.49	1	0
	Water	1.68 ± 0.16	< 0.001	> 99.9
PP	Intercept	0 ± 0.01	1	0
	PE	0.26 ± 0.09	< 0.01	99.6
	PP	0.06 ± 0.24	0.815	18.5
	PS	0 ± 0.23	1	0
	PVC	0 ± 0.35	1	0
	Water	1.67 ± 0.11	< 0.001	> 99.9
PS	Intercept	0.01 ± 0.003	< 0.01	99.0
	PE	0 ± 0.02	1	0
	PP	0 ± 0.06	1	0
	PS	0.31 ± 0.06	< 0.001	> 99.9
	PVC	0 ± 0.09	1	0
	Water	1.26 ± 0.03	< 0.001	> 99.9
PVC	Intercept	0.02 ± 0.002	< 0.001	> 99.9
	PE	0 ± 0.02	1	0
	PP	0.07 ± 0.05	0.166	83.4
	PS	0 ± 0.05	1	0
	PVC	0.33 ± 0.07	< 0.001	> 99.9
	Water	1.08 ± 0.02	< 0.001	> 99.9

*: If the coefficient is < 0.01, it is assumed to be zero.

The validity of the prediction algorithm was first evaluated by examining the spectral fitting residuals, defined as the difference between the predicted and the observed spectra. The prediction residuals, arising from incompleteness of the algorithm or the measurement uncertainties, are in general comprised < 10% of the observational data, which is small enough for a valid simulation. These results indicated that the algorithm was successful even under relatively optically thick conditions when the plastics were submerged in water, although optically thin cases tend to be more easily reproduced in principle. The coefficient of each target polymer was the highest among the coefficients of the other materials, with the exception of water, in the regression model (Table 1). In comparison, for PP in water, the contribution from water was dominant, while the contributions from PE overwhelmed that of PP. This is due to the similarity of the spectra of PP and PE in both the dry and submerged states within the 1100–1300 nm range (Fig. 2). Consequently, the featured spectra of PP in water at a depth of 2.5 mm were not reproduced.

**Figure 4:**
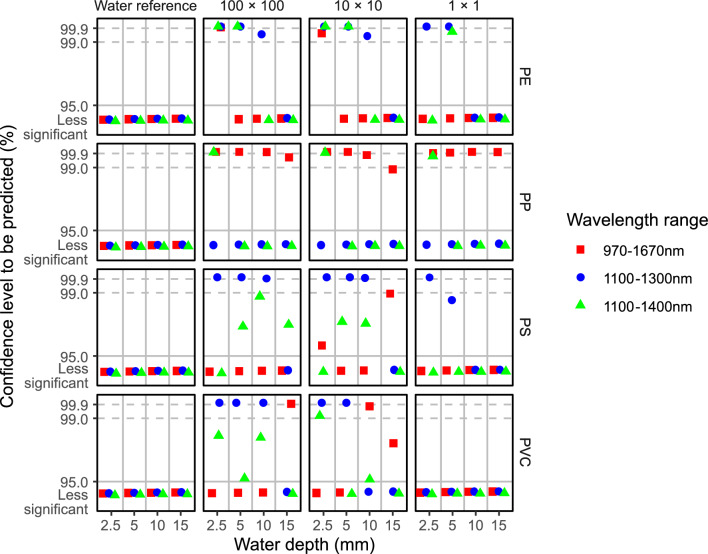
Predictability of four common polymers when submerged in water. The predictability is expressed as a confidence level as a function of the detection area size (100 × 100 pixels, 10 × 10 pixels, and 1 × 1 pixel), the water depth (2.5 mm, 5 mm, 10 mm, and 15 mm), and the wavelength range (1100–1300 nm, 1100–1400 nm, and 970–1670 nm). A water reference area (10 × 10 pixels) was also included for each polymer. Confidence levels below 95% are marked as less significant.

The predictability for the four common polymers in water was investigated further with respect to the detection area size, the water depth, and the wavelength range (Fig. 4). For each of the combined factors, a polymer in water was regarded as predictable if the confidence level value (*c*) of the target polymer was the highest among all the driving polymers and if it was > 95%. Here, *c* is defined as 1 – *p,* where *p* is the probability to evaluate the significance of the coefficient of each driving polymer. In addition to the three sizes of the plastics in the water, regions of interest covering water-only pixels (the white area in Fig. S1d) in each image were included in the predictions to serve as nonplastic references. The spectra of the areas without plastic pieces in each image did not show relationships with the spectra of any of the four common polymers in water, indicating that the plastic polymers were only recognized when they were present.

Among the different polymers in the water, PE, PS, and PVC showed predictability over broad size and submerging water depth ranges within the 1100–1300 nm wavelength range, while the best wavelength range for prediction of PP in water was 970–1670 nm (Fig. 4); this is consistent with previous studies^29,32^. Specifically, PE and PS could be predicted when submerged by 2.5–5 mm in the 1100–1300 nm wavelength range, even when the detection area size was reduced from 100 × 100 pixels (16.0 × 16.0 mm^2^) to 1 × 1 pixel (0.16 × 0.16 mm^2^). PVC submerged in water to depths of 2.5–5 mm could be predicted for the 100 × 100 pixel and 10 × 10 pixel sizes over the 1100–1300 nm range, but it could not be predicted for the 1 × 1 pixel size, indicating that spatial averaging aided the analysis. The predictability of PE, PS, and PVC when submerged in deeper water (≥ 10 mm) decreased when the featured spectra were largely subject to interference from the water. In comparison, PP submerged in water was predictable in the 970–1670 nm wavelength range, rather than the 1100–1300 nm range. This predictability remained even for the 1 × 1 pixel size and under 15 mm of water. The featured spectra of PP spanned broadly over the 970–1670 nm range under these small size and greater water depth conditions, but they were not perceivable in the 1100–1300 nm range when using the regression algorithm.

### Predictability of five minor polymers in water

**Figure 5:**
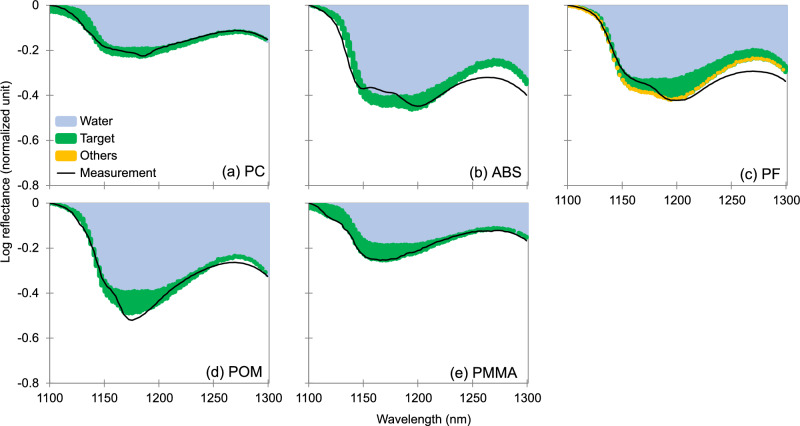
Separated contributions to the spectra of five minor submerged polymers. Reflectance spectra of (**a**) PC, (**b**) ABS, (**c**) PF, (**d**) POM, and (**e**) PMMA in 2.5 mm of water were composed of contributions from each target polymer, the other polymers, and water, and were compared with the measurements. The logarithmic reflectance obtained after normalization was performed by subtracting the maximum value of each polymer in the 1100–1300 nm wavelength range on an area of 100 × 100 pixels are shown.

Five polymers existing as relatively less abundant environmental pollutants, PC, ABS, PF, POM and PMMA, were investigated for their predictability when submerged in water as well. The reflectance spectra of PC, POM, and PMMA at a water depth of 2.5 mm on an area size of 100 × 100 pixels were predominantly contributed by each target polymer within the 1100–1300 nm range (Table S1). Each of these featured spectra could be largely retained after the interference from water was eliminated (Fig. 5a, d, and e). In comparison, the featured spectra of ABS and PF at around 1150 nm and 1200 nm were both largely flattened by water when these plastics were submerged (Fig. 2), although the contributions from the target polymers were again the largest when compared with the other polymer types. Statistically, this in turn led to inadequate reproductions of the spectra by the regression model (Fig. 5b and c).

**Figure 6:**
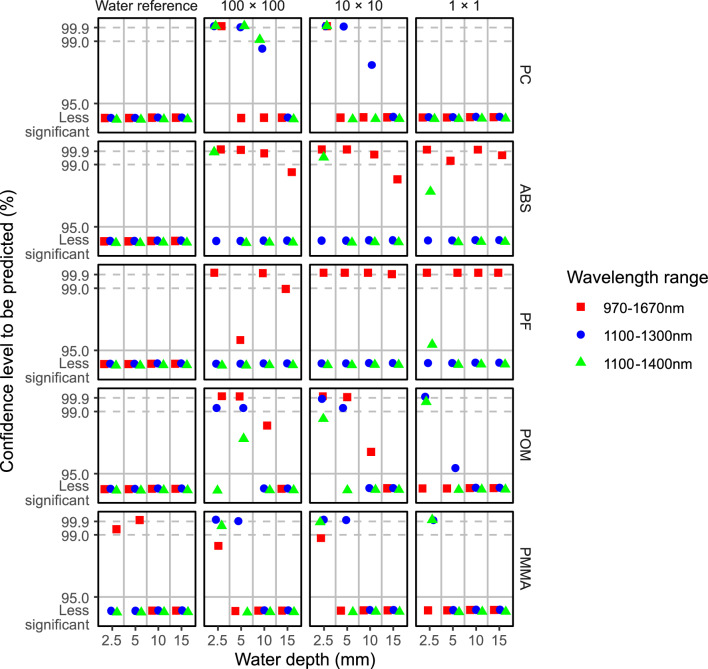
Predictability of five minor polymers when submerged in water. The predictability is expressed as a confidence level as a function of the detection area size (100 × 100 pixels, 10 × 10 pixels, and 1 × 1 pixel), the water depth (2.5 mm, 5 mm, 10 mm, and 15 mm), and the wavelength range (1100–1300 nm, 1100–1400 nm, and 970–1670 nm). A water reference area (10 × 10 pixels) was also included for each polymer. Confidence levels below 95% are marked as less significant.

When the analysis was extended to broader size and water depth ranges, the predictability for the five minor polymers was highly dependent on the wavelength range (Fig. 6). In the 1100–1300 nm wavelength range, POM and PMMA submerged in water to a depth of 2.5 mm could be predicted down to the 1 × 1 pixel size, while the 10 × 10 pixel size was the prediction limit for PC in water. For POM and PMMA, 5 mm was the water depth limit for prediction in the 1100–1300 nm wavelength range, while PC could be predicted in the same wavelength range when submerged in water up to a depth of 10 mm. In comparison, ABS submerged in 2.5 mm of water could be predicted over the 1100–1400 nm range down to 1 × 1 pixels, but if the wavelength range was extended to 970–1670 nm, the signal could be reproduced even when it was submerged in 15 mm of water. Similarly, the 970–1670 nm range was also appropriate for simulation of PF in water. It is worth nothing that PMMA in water was falsely simulated as being predictable by the nonplastic area in the 970–1670 nm wavelength range for 2.5–5 mm water depths. Under these conditions, the signals of the water pixels and the polymer pixels were not differentiable by the model. These results indicated that rather than the wide wavelength range, the 1100–1300 nm and 1100–1400 nm wavelength ranges were more suitable for prediction of PMMA in water.

## Discussion

### Potential for detection of plastics in water under benchtop conditions

Elimination of the interference from water is an essential technical requirement for direct detection of submerged plastics when using the hyperspectral imager. In the optimized detection environment on a benchtop system, our results indicated that the interference from water could be separated using a linear regression model after logarithmic conversion for the nine polymers when submerged in surface water. This indicates that a drying pretreatment process of environmental samples before hyperspectral analysis could be skipped, contributing to an improved efficiency. It should be noted that the prediction algorithms are dependent on an empirical selection of driving variables. For prediction of each of these polymers in water, we deliberately included not only the target polymer, but also other polymer types in each case to validate the arbitrariness of the algorithm. Each of the target polymers was found to be the dominant driving variable for prediction of its spectra in water, thus indicating the robustness of the algorithm. Nevertheless, because environmental plastics are often mixed with nonplastic debris, e.g., glass, stone, sand, and wood, further studies to include the associated variables in the algorithm will be required. Instead of pure water, investigations based on sea water would be another future work.

The predictability of the polymers when submerged in surface water up to depths of 15 mm was investigated. The degree of interference caused by water increased rapidly as the submerged depth increased beyond 10 mm, where the linearity might not be obtained. This indicated that although polymers with low densities (e.g., PE, PP, and PS) that are suspended in fresh water and/or seawater are more readily predictable, polymers with densities higher than that of water (e.g., PVC, PC, POM, ABS, PF, and PMMA) may sink gravitationally to depths at which their reflectance spectra could not be captured directly using a hyperspectral imager. When using the current methods, polymers in the wet state or with water layers on top of them could be detected directly under benchtop hyperspectral detection conditions, and our results suggested that a water depth of ≤ 10 mm is optimal. Moreover, polymers with dimensions down to 1.6 × 1.6 mm^2^ (PVC and PC) and 0.16 × 0.16 mm^2^ (PE, PP, PS, ABS, PF, POM and PMMA) could be detected. In future work, it is expected that the minimum detection size will be improved further when the system can be optimized to detect particles on the micrometer scale.

The optimal wavelength ranges for prediction of the spectra of the plastics in water varied among the polymers. For PE, PS, PVC, PC, POM, and PMMA, 1100–1300 nm was the most favorable range for prediction of each of their spectra when they were submerged, attributing to little interference by water. For PP and ABS, the 1100–1400 nm range was best for the prediction of each of their spectra in water up to a 2.5 mm depth, while a broader range of 970–1670 nm was necessary for the predictions when they were submerged in deeper water. The 970–1670 nm range was also most favorable for the prediction of PF in water. Tasseron et al. reported that an average spectral signature for polymers comprising high-density PE, low-density PE, PP, PS, and polyethene terephthalate showed two distinct absorption peaks at 1215 nm and 1410 nm with the aim of separating the interference produced by water^34^. In comparison, we have presented comprehensive results that cover a variety of polymers and water depths. These results will form a fundamental basis for further development of equipment and identification algorithms to detect plastics in aquatic environments using hyperspectral imagers.

### Implications for aquatic plastic detection aboard airborne and satellite platforms

The hyperspectral imager’s capability for detection of aquatic plastics in the field is dependent on factors including photographic conditions (e.g., illumination conditions, water vapor), the submerging water conditions (depth, current, turbidity), and the target plastics (polymer type, size, color, adhesiveness/mixing with impurities/substrates, weathering state)^21,45–49^. In this work, the possibility of direct sensing of plastics when submerged in water was investigated under benchtop experimental conditions. The results firmly support the hypothesis that the interference caused by water in the reflectance spectra of plastics can be separated, which is referable for further development of algorithms to detect submerged plastics present at non-prescribed water depth in the field. Specifically, featured wavelength ranges required to sense different polymers when submerged in water were identified. From the viewpoint of polymer composites, our findings indicate that when an imager that covers the 1100–1300 nm wavelength range is used, there is a potential for up to 43% (61% for an imager covering the 1100–1400 nm range) of the environmental plastics suspended in surface water to be detected directly with reference to the polymer-specific shares of plastic waste^44^. The fact that the analysis was successful in the narrow wavelength range implies the potential to use low-cost light-emitting diode devices as the light sources in the future as well.

With the aim of detecting plastics from space using hyperspectral sensors, several satellite products have been recently launched. The PRecursore IperSpettrale della Missione Applicativa (PRISMA) mission carries a hyperspectral sensor with spectral coverage of 400–2500 nm and resolution of < 15 nm, which is fitted well to the sensitive range for plastics^50^, with a relatively coarse ground resolution of 30 m. Sensors with similar spectral coverage and ground resolutions were launched aboard the Environmental Mapping and Analysis Program (EnMAP) mission (420–2450 nm; 30 m) and the Gaofen-5 mission^39,51–53^. The Hyperspectral Imager Suite (Hisui) sensor that was launched to the International Space Station in 2019 provided global coverage in addition to the coverage features mentioned above (400–2500 nm, 20–30 m)^54^. Our work provides the prospect of detection of large-scale accumulated plastics with respect to their polymer-specific levels in the ocean using these satellites. Furthermore, our results for the most sensitive spectral ranges of the different polymers will contribute to the development of next-generation, low-cost, multi-spectral sensors with improved ground resolution. Our findings will also provide information toward the development of an atmospheric correction algorithm to eliminate the interference from water vapor.

## Conclusions

Light absorption by water in the NIR-SWIR range has been an obstacle to direct detection of plastics in water using hyperspectral imagers. We investigated the reflectance spectra of plastics when submerged in surface water and developed algorithms to eliminate the interference from water. We first collected hyperspectral images in the 900–1700 nm wavelength range for nine polymers submerged in water to millimeter-scale depths to simulate floating conditions. Their reflectance spectra were interfered by the absorbance of water when submerged, particularly when the depth was > 10 mm. We also developed algorithms to account for composite reflectance spectra of plastics in water with respect to the separate contributions from the polymers and the water. The probability of eliminating water interference from the submerged plastics was then evaluated regarding the polymers, the wavelength ranges, the water depths, and the plastic sizes. A wavelength range of 1100–1300 nm was sufficient to eliminate the interference from water for PE, PS, PVC, PC, POM, and PMMA when submerged. In comparison, a broader range of 970–1670 nm was proposed to eliminate the interference from water for PP, ABS, and PF. Regarding the water depth, polymers submerged in water not deeper than 10 mm tend to be detected readily. The methods allow for detection of submerged PE, PP, PS, ABS, PF, POM, and PMMA in the small size range of approximately 0.16 × 0.16 mm^2^ with potential for further improvement. Our findings provide fundamental information for direct detection of plastics when submerged in water, not only for benchtop systems, but also for applications aboard airborne and satellite platforms.

## Materials and methods

### Hyperspectral imaging system

We modified a commercially available benchtop hyperspectral imaging system to sense plastics with sizes ranging from micrometer to centimeter scales^26^. Briefly, a two-dimensional InGaAs array detector with a pixel size of 15 µm was incorporated into a hyperspectral imager (Pika NIR-640, Resonon Inc., Bozeman, Montana, USA). The imager had wavelength coverage of 900–1700 nm with spectral resolution of 5.6 nm. A lens (SR2343-A01, StingRay Optics Inc., Keene, New Hampshire, USA) that was optimized for the same wavelength range with a focal length of 25 mm and a field of view of 21.7° was used for the photography. With 640 spatial channels, the imager was mounted on a frame above the translation stage to perform push-broom line scanning (Fig. S1a, b). A symmetrical pair of convergent light NIR-SWIR lamps (LN-200CIR, CCS Inc., Kyoto, Japan) with wavelength coverage of 400–2500 nm was installed on the left and right sides of the imaging system to provide stable illumination with low radiant heat. Image acquisition was controlled using Spectronon Pro software (version 2.5, Resonon Inc., Bozeman, Montana, USA) on a computer, where the data were saved as three-dimensional cubes. With the modified system, microplastics as small as 100 µm could be rapidly detected.

### Setup of plastics in water and the hyperspectral image acquisition

A total of 45 NIR-SWIR hyperspectral images were acquired for nine common polymers composed of authentic plastics (Table 2) in the dry state and when submerged to four water depths (2.5 mm, 5 mm, 10 mm, and 15 mm) (Fig. S2). For each polymer, a plate with dimensions of 50 mm × 50 mm × 1 mm was adhered to a stainless-steel petri dish (*φ* 75 × 20 × 0.6 mm^3^; As One Inc., Japan) using glue (Fig. S1c), where a small amount of glue (ca. 0.05 ml) was applied to each corner of the plate. Distilled water (Fujifilm Wako Pure Chem. Corp. Osaka, Japan) with volumetric amount corresponding to each of the target submerging depths subtracting the volume of plastic plate was then added to the petri dish, i.e., 12.2 cm^3^, 22.7 cm^3^,43.7 cm^3^ and 64.6 cm^3^ for submerging depths of 2.5 mm, 5 mm, 10 mm and 15 mm, respectively. Because the corner regions were not selected for the subsequent image processing, no effect on the spectra was expected from the glue. During image acquisition, the distance between the lens and the stage was set at 30 cm, the frame rate was set at 25 s^–1^, the scan speed was set at 0.3969 cm s^–1^, and the scanning distance was set at 600 lines. Under these conditions, the nominal pixel size for the images was ca. 0.16 mm. Before observing the plastics, the imager was first corrected for the dark state (reflectance = 0) when the lens is covered with a cap, and for the reference (reflectance = 1) state with a white target (nominal reflectance: 99%; SRT-99–100, Labsphere, Inc., North Sutton, New Hampshire, USA).

**Table 2:** Authentic plastic polymers*.

Abbreviation	Full name	Color
PE	Polyethylene	Natural (semitransparent)
PP	Polypropylene	Natural
PS	Polystyrene	Transparent
PVC	Polyvinyl chloride	Transparent
PC	Polycarbonate	Transparent
ABS	Acrylonitrile butadiene styrene	Natural
PF	Phenol formaldehyde	Brown
POM	Polyacetal	Natural
PMMA	Polymethyl methacrylate	Transparent

*All these polymers were obtained from As One Corp., Osaka, Japan, in plates with dimensions of 100 mm × 100 mm × 1 mm, and were cut to dimensions of 50 mm × 50 mm × 1 mm for the study.

### Image processing

Each of the images obtained contains three-dimensional information in the form of 640 samples (horizontal direction in Fig. S1c) × 600 lines (vertical direction) × 328 wavelength channels. The regions of interest in the center right area of each image were selected, covering 100 × 100 pixels (corresponding to an area of 16.0 × 16.0 mm^2^), 10 × 10 pixels (1.60 × 1.60 mm^2^), and 1 pixel (0.16 × 0.16 mm^2^), respectively (Fig. S1d). An area covering water only (10 × 10 pixels) was also selected in the left edge area of each image for comparison. The mean reflectance spectra were then calculated over each of the selected regions (e.g., mean spectra of 100 × 100 pixels in Fig. 2). To enable prediction of the reflectance spectra of the plastics in water, the spectra were first converted into their logarithms (Fig. S3). For each polymer at each water depth, the logarithmic spectrum was offset further by subtracting the maximum value along the wavelength range, which was to represent the wavelength-independent reflectance of the stainless steel without the influence of plastic absorbance (Fig. S4).

### Prediction algorithm for four common polymers

Four common polymers (PE, PP, PS and PVC) submerged in water were first investigated on the predictability of their reflectance spectra. An ideal prediction for one polymer in water is that its spectrum is contributed purely by the spectrum of that polymer in the dry state and that of water to the same depth. However, to examine the validity of the proposed algorithm, other polymers were included simultaneously to act as driving parameters. The rationale is that the algorithm is valid if the spectrum of one polymer in water is first contributed by the spectrum of that polymer itself when compared with the other polymers.

The processed spectra were then subjected to a multiple linear regression model, in which an adaptive least-squares algorithm was applied^55^. In the model, with the aim of predicting the target variable (i.e., the spectrum of each polymer in the water in the study), the empirical constants (coefficients) of each of the driving parameters (the spectra of the contributing polymers in the dry state and water) were optimized through minimization of the nonlinear residuals. For each polymer at each water depth *d*, the logarithms of the normalized reflectance spectra Log *R*_*norm,d*_(*λ*) were fitted as a linear combination of the contributions from the absorbing (and thus force-reflecting) compounds (i.e., water and polymers) as shown by Eq. (1):

1 $$\begin{aligned} {\text{Log R}}_{{{\text{norm}},{\text{ d}}}} { }\left( {\uplambda } \right) & = {\text{ a }} + {\text{ b }} \times {\text{ Log Water}}_{{{\text{norm}},{\text{ d}}}} \left( {\uplambda } \right) + {\text{ c }} \times {\text{ Log PE}}_{{{\text{norm}},{\text{ dry}}}} \left( {\uplambda } \right) + {\text{ d }} \\ & \;\;\;\; \times {\text{ Log PP}}_{{{\text{norm}},{\text{ dry}}}} { }\left( {\uplambda } \right) + {\text{ e }} \times {\text{ LogPS}}_{{{\text{norm}},{\text{ dry}}}} { }\left( {\uplambda } \right) + {\text{ f }} \times {\text{ Log PVC}}_{{{\text{norm}},{\text{ dry}}}} \left( {\uplambda } \right) \\ \end{aligned}$$

where Log *Water*_*norm,d*_(*λ*) represents the logarithm of normalized reflectance spectrum of water at the corresponding depth and wavelength *λ*, and Log *PE*_*norm,dry*_(*λ*), Log *PP*_*norm,dry*_(*λ*), Log *PS*_*norm,dry*_(*λ*), and Log *PVC*_*norm,dry*_(*λ*) represent the logarithms of the normalized reflectance spectra of each polymer in the dry state. The lower bound of the fitting coefficients (a–f) was designated to be zero to provide a more realistic fit.

For each fitting, to provide the best predictions for the target polymer in water, the coefficients of the driving polymers were simulated along with the standard errors and the corresponding *p* values (i.e., the statistical probability that the estimated coefficient occurred by chance). To illustrate the contributions from each polymer more intuitively, the confidence level (*c*) was then expressed as 1 − *p*. Specifically, a high *c* value here indicates a high contribution from the polymer to the target polymer in the water.

For each polymer at each water depth, the predictability of the plastics was investigated with regard to the wavelength ranges and pixel sizes used. Three wavelength ranges were screened out for prediction of the reflectance of the plastics, covering 970–1670 nm, 1100–1300 nm, and 1100–1400 nm, at the previously mentioned areas of 100 × 100 pixels, 10 × 10 pixels, and 1 pixel.

### Prediction algorithm for five minor polymers

Five polymers that exist as relatively less abundant environmental pollutants, comprising PC, ABS, PF, POM and PMMA, were also investigated in terms of their predictability when submerged in water in a similar manner to the four common polymer types. Moreover, given the common nature of the four dominant polymer types in the environment, they were also included to validate the interference effect on the prediction of the five minor polymers. The prediction algorithm was thus expressed as shown in Eq. (2):

2 $$\begin{aligned} {\text{Log Poly}}\_{\text{Minor}}_{{{\text{norm}},{\text{ d}}}} { }\left( {\uplambda } \right) & = {\text{ a }} + {\text{ b }} \times {\text{ Log Water}}_{{{\text{norm}},{\text{ d}}}} \left( {\uplambda } \right) + {\text{ c }} \\ & \;\;\;\; \times {\text{ Log PE}}_{{{\text{norm}},{\text{ dry}}}} \left( {\uplambda } \right) + {\text{ d }} \times {\text{ Log PP}}_{{{\text{norm}},{\text{ dry}}}} { }\left( {\uplambda } \right) + {\text{ e }} \times {\text{ LogPS}}_{{{\text{norm}},{\text{ dry}}}} { }\left( {\uplambda } \right) \\ & \;\;\;\; + {\text{ f }} \times {\text{ Log PVC}}_{{{\text{norm}},{\text{ dry}}}} \left( {\uplambda } \right) + {\text{ g }} \times {\text{ Log Poly}}\_{\text{Minor}}_{{{\text{norm}},{\text{ dry}}}} \left( {\uplambda } \right) \\ \end{aligned}$$

where Log *Poly_Minor*_*norm,d*_ represents the logarithm of the normalized reflectance spectra of each of the five minor polymers, Log *Water*_*norm,d*_(*λ*) represents the logarithm of the normalized reflectance spectra of water at the corresponding depth and wavelength *λ*, and Log *PE*_*norm,dry*_(*λ*), Log *PP*_*norm,dry*_(*λ*), Log *PS*_*norm,dry*_(*λ*), Log *PVC*_*norm,dry*_(*λ*), and Log *Poly_Minor*_*norm,dry*_(*λ*) represent the logarithms of the normalized reflectance spectra for the four common polymers and the target minor polymer, respectively, in the dry state. The predictability of each polymer at each water depth was then investigated in a similar manner to that of the four common polymers.

### Data processing environment and packages

Image processing, plotting, and simulations were conducted in an R language environment (version 4.2.1), where “hyperSpec”, “ggplot” and “nls2” (“port” algorithm) packages were used.

### Supplementary Information

Supplementary Information.

## Data Availability

All data are available from the corresponding authors upon request.
