# Supplementary material for: Eliminating the interference of water for direct sensing of submerged plastics using hyperspectral near-infrared imager
Source: Sci Rep. 2023 Oct 6;13:15991. doi: 10.1038/s41598-023-39754-7 (PMC10558484; doi:10.1038/s41598-023-39754-7)
Supplement: Supplementary file 1 — Supplementary Information. [file 41598_2023_39754_MOESM1_ESM.pdf]

Supplementary information for

Eliminating the interference of water for direct sensing of submerged plastics using hyperspectral near-infrared imager

Chunmao Zhu[1*], Yugo Kanaya[1]

[1]Research Institute for Global Change, Japan Agency for Marine-Earth Science and Technology (JAMSTEC), Yokohama, Kanagawa, 2360001, Japan

*Corresponding author

Email: chmzhu@jamstec.go.jp
Tel.: +81-45-778-5365

The file contains Table S1 and Figures S1-S4.

Table S1. Prediction parameters of 5 minor polymers in 2.5-mm-depth water in the 1100–1300 nm wavelength range on a size area of 100 × 100 pixels.

Target polymer	Driving variable	Coefficient	*p* value	Confidence level (*c*, %)
	Intercept	0.01 ± 0.003	< 0.01	99.8
	PC	0.46 ± 0.11	< 0.001	> 99.9
	PE	0 ± 0.02 *	1	0
PC	PP	0 ± 0.06	1	0
	PS	0 ± 0.10	1	0
	PVC	0 ± 0.09	1	0
	Water	0.98 ± 0.04	< 0.001	> 99.9
	Intercept	0 ± 0.02	1	0
	ABS	0.31 ± 0.27	0.26	74.0
	PE	0 ± 0.13	1	0
ABS	PP	0 ± 0.39	1	0
	PS	0 ± 0.65	1	0
	PVC	0 ± 0.71	1	0
	Water	2.06 ± 0.18	< 0.001	> 99.9
	Intercept	0 ± 0.01	1	0
	PF	0.43 ± 0.50	0.39	60.7
	PE	0.05 ± 0.10	0.61	38.8
PF	PP	0 ± 0.44	1	0
	PS	0 ± 0.27	1	0
	PVC	0 ± 0.44	1	0
	Water	1.69 ± 0.16	< 0.001	> 99.9
	Intercept	0 ± 0.01	1	0
	POM	0.33 ± 0.12	< 0.01	99.2
	PE	0 ± 0.06	1	0
POM	PP	0 ± 0.16	1	0
	PS	0 ± 0.16	1	0
	PVC	0 ± 0.38	1	0
	Water	2.00 ± 0.12	< 0.001	100
	Intercept	0 ± 0.004	1	0
	PMMA	0.54 ± 0.11	< 0.001	> 99.9
	PE	0 ± 0.02	1	0
PMMA	PP	0 ± 0.08	1	0
	PS	0 ± 0.10	1	0
	PVC	0 ± 0.13	1	0
	Water	0.95 ± 0.05	< 0.001	> 99.9

*: If the coefficient is < 0.01, it is assumed as 0.

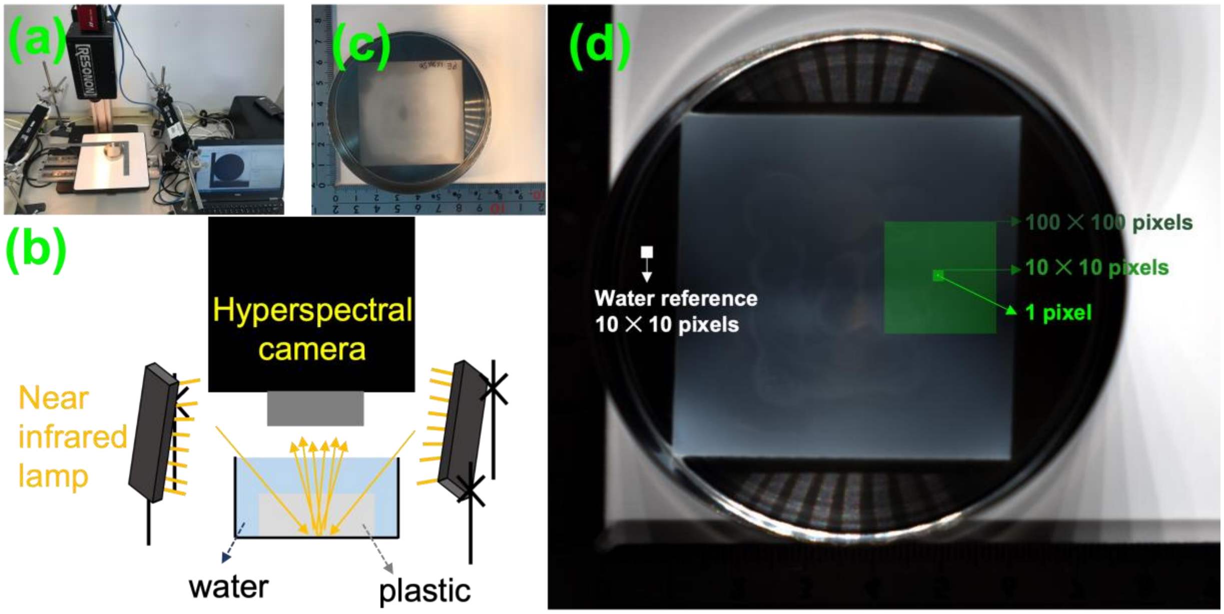

Figure S1. Acquisition of hyperspectral imageries of plastics in water. (a) Improved hyperspectral imaging system with a NIR camera (Pika NIR-640) and a pair of NIR light sources. (b) Sketch of the reflectance measurement mode. (c) Example photo of authentic plastic plate (50 mm × 50 mm × 5 mm) taken by a visible camera. (d) Example pseudo imagery acquired by the system composed at the 1100 nm, 1300 nm and 1500 nm. The green shades show the regions of interests for predicting reflectance spectra of plastics in water, where the pixel size is ca. 0.16 mm. An area without plastic was selected as water reference (white shade).

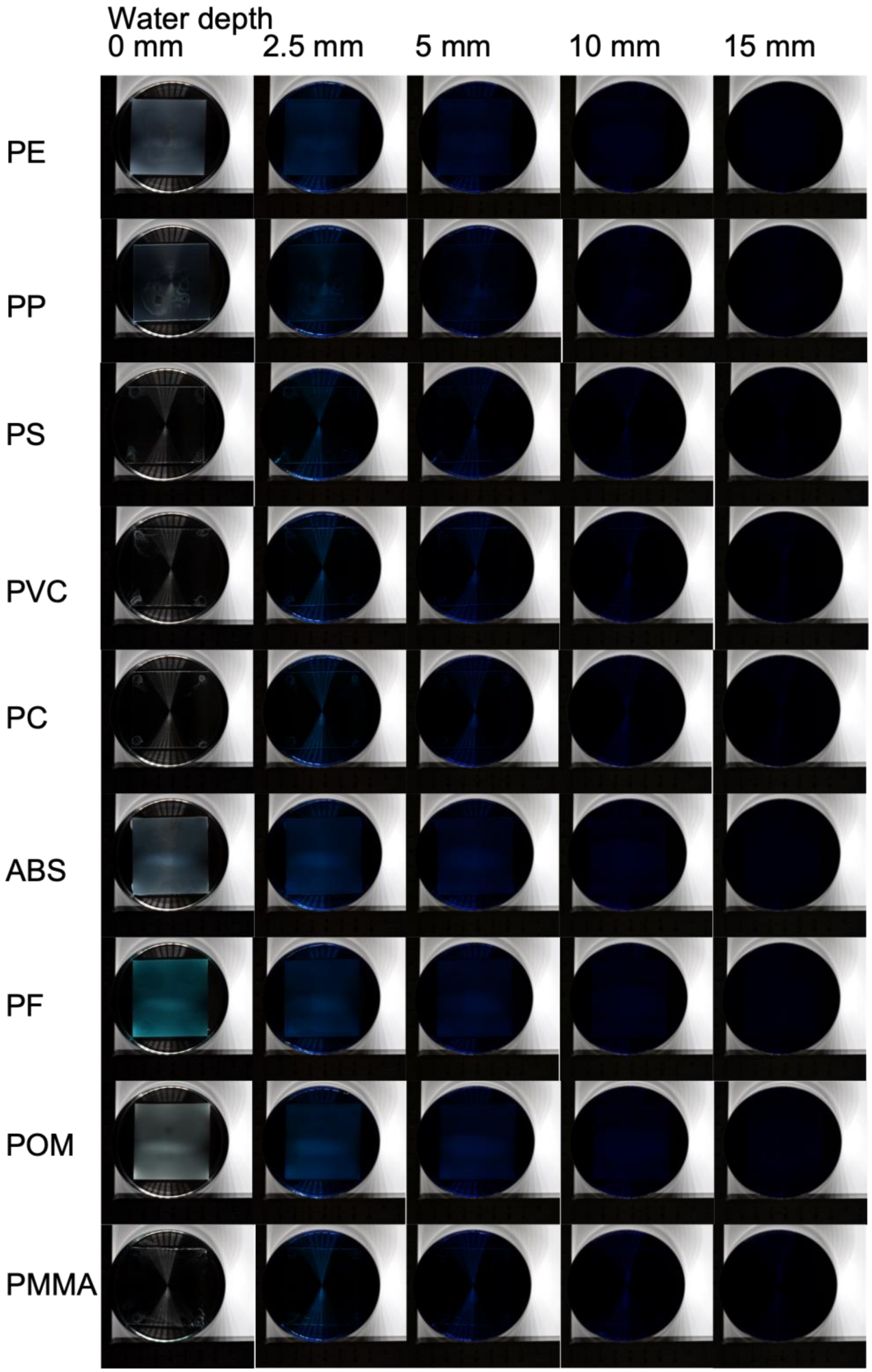

Figure S2. Hyperspectral imageries of 9 polymers in the dry state (0 mm) and submerged in 4 water depths (2.5 mm, 5 mm, 10 mm and 15 mm), respectively. Pseudo imageries composed at the 1100 nm, 1300 nm and 1500 nm were shown.

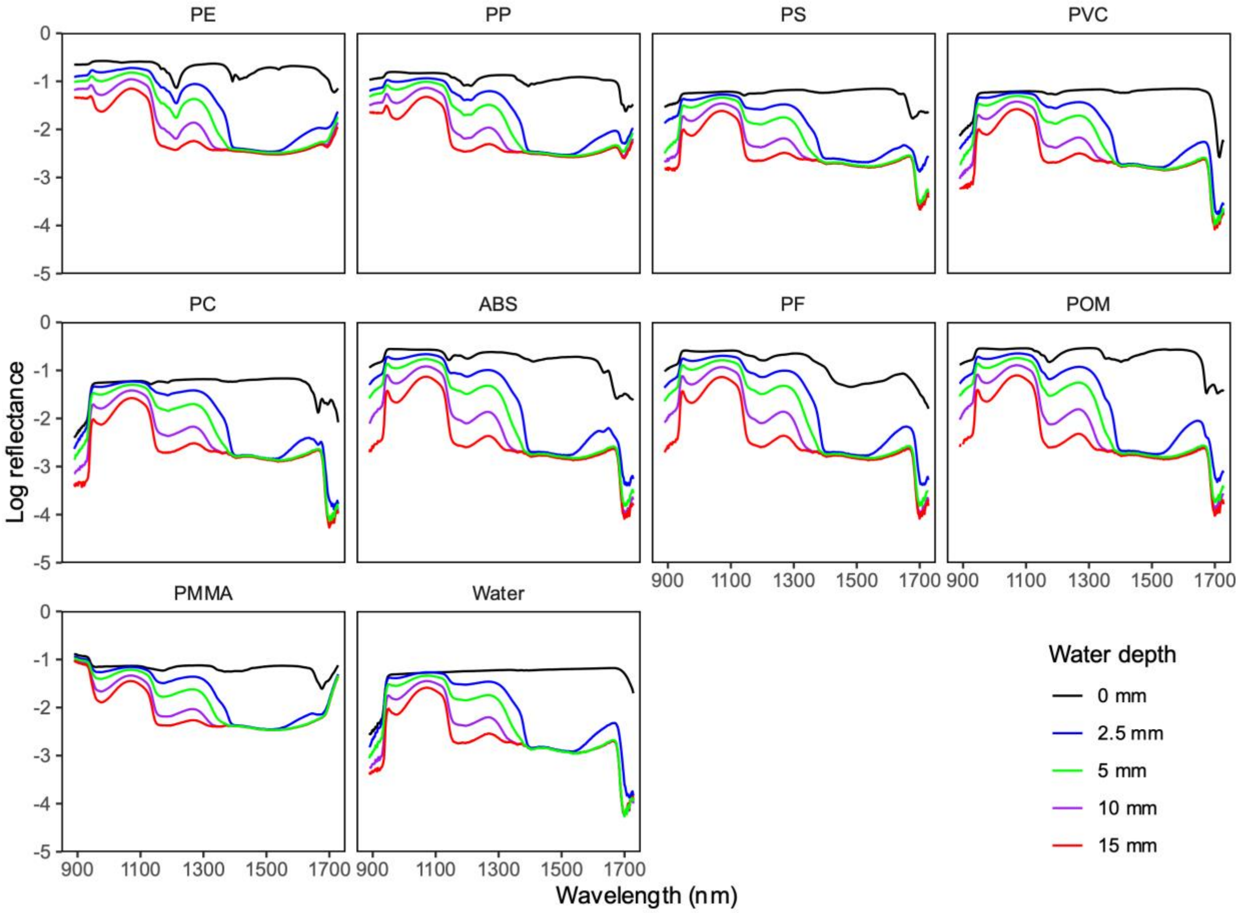

Figure S3. Logarithmic reflectance spectra of 9 common polymers in the dry state (0 mm) and submerged in water (2.5 mm, 5 mm, 10 mm and 15 mm depths), respectively.

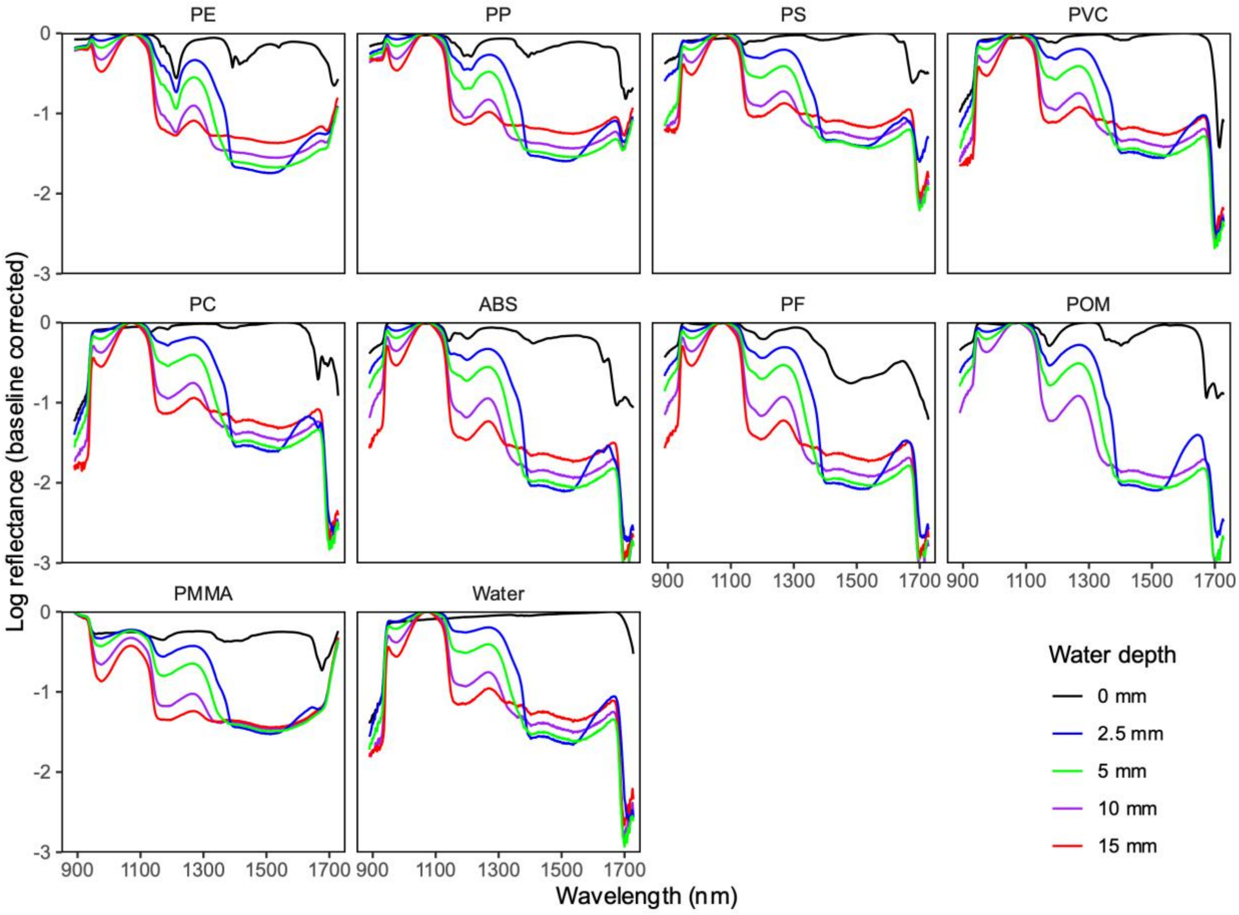

Figure S4. Normalized logarithmic reflectance spectra of 9 common polymers in water.
